# Supplementary material for: Maternal Melatonin Contributes to Offspring Hair Follicle Development Through Transcriptional Regulation of the AP-1 Complex and MAPK Pathway
Source: Int J Mol Sci. 2025 Feb 24;26(5):1952. doi: 10.3390/ijms26051952 (PMC11900504; doi:10.3390/ijms26051952)
Supplement: Supplementary file 1 [file ijms-26-01952-s001.zip › ijms-3409095-supplementary.pdf]

International Journal of
Molecular Sciences

MDPI

Supplementary information

1. Supplementary Methods

1.1. RNA-seq analysis

The TRIzol method was used to extract total RNA from skin samples.RNA quality determination and RNA-seq analyses were performed by NovogeneInc. (Beijing, China). The reference genome and rabbit gene annotation files were downloaded from Ensembl (https://asia.ensembl. org/Oryctolagus_cuniculus/Info/Index), andHISAT2software (version 2.0.5)was used to align the clean paired-end reads to the reference genome. The RNA-seq data presented in this study have been submitted to the NCBI (SubmissionID: SUB12096610).Using HTSeq(version 0.6.1), we calculated the number of counts and reads associated with the mapped genes. We also calculated the fragments per kilobase per million mapped (FPKM) and read counts mapped to thegenes based on the gene length. Analysis of DEGsbetween the MT and CR groups was performed using the DESeq2 package of R (version 1.20.0), which also provides statistical analyses for DEGs. P-values were adjusted based on Benjamini and Hochberg's false discovery rate control method. Genes with adjusted P-values< 0.05 from DESeq2 and |log2foldchange| > 0.5 were considered significantly differentially expressed. The ClusterProfilersoftware(version 3.4.4)was used for the Gene Ontology (GO) enrichment analysis and Kyoto Encyclopedia of Genes and Genomes (KEGG) pathway analysis of the DEGs. The GO analysis mainly includes cellular components(CC) and biological processes (BP). The DEGs in the KEGG enrichment pathway were considered significantly enriched when they had P-values<0.01.

1.2. Topology algorithm methods

To identifythe hub genes,we used 12 algorithms, including Degree, Edge Percolated Component (EPC), Maximum Neighborhood Component (MNC), Density of Maximum Neighborhood Component (DMNC), Maximal Clique Centrality (MCC), Bottleneck (BN), EcCentricity, Obligatory, Radiality, Betweenness, Clustering Coefficient, and Stress.The top 30 DEGs under went further selection, and thecalculation methods were used as previously reportedbyChinet al. [58].

1.3. Quantitative real-time reverse transcription-polymerase chain reaction (qRT-PCR)

The *ACTB* gene was used as the reference gene. The primer sequences used to amplify these genes were obtained from NCBI (https://www.ncbi.nlm.nih.gov) and are listed in Supplementary Table S2. RNA isolation, reverse transcription, qRT-PCR determination, and relative gene expression calculation were performed as our described previously[25].

1.4. Western blotting

Rabbit skin tissues were fully ground in lysis buffer, followed by ultrasonic lysis. The skin tissue homogenate was then centrifuged, the supernatantwas collected, and the total protein concentration was determined. The proteins were separatedvia sodium dodecyl sulfate-polyacrylamide gel electrophoresis (SDS-PAGE) and electrotransferred to a polyvinylidene difluoride (PVDF) membrane (300mA, 30 min).Normalization was performed by stripping and saturating the blots with an anti-actinantibody. Immunoreactive bands were visualized using enhanced chemiluminescence, and protein expression was quantified using Quantity One software (version 4.6.6).

 https://doi.org/10.3390/ijms26051952

1.5. Metabolic profiling

After grinding 100 mg of skin tissue with liquid nitrogen, the homogenate was resuspended in prechilled 80% methanol and 0.1% formic acid by vortexing. After centrifugation and dilution, the supernatant was subjected to liquid chromatography and mass spectrometry(LC/MS) as described previously [59]. Raw UHPLC-MS/MS data were processed using Compound Discoverer 3.1 software to align peaks, pick peaks, and quantify each metabolite[60]. Metabolomics Raw mass spectrometry data were uploaded to Metabolights (Study ID: MTBLS6009). Orthogonal partial least squares-discriminant analysis(OPLS-DA) wasperformed using MetaboAnalyst software (version 5.0). *P*-values were calculated using univariate analysis (*t*-test). Metabolites with VIP > 1 and *P*-values< 0.01 were considered differentially expressed metabolites (DEMs). MetaboAnalyst software was also used for differential metabolite enrichment and topological analysis [61]. Debiasedsparse partial correlation (DSPC) was then conducted to identify correlations between differential metabolites[60].

1.6. Terminal deoxynucleotidyl transferase-mediated dUTP-nick end labeling (TUNEL) assay

Skin tissue slices and paraffin sectioning wereperformed as previously described[25].Proteinase K working (75 μg/ml) solution was added to cover the sections, which were incubated for 25 min at 37°C. The sections were then washed thrice with PBS (pH 7.4) and placed on a rocker device. Next, terminal deoxynucleotidyl transferase and luciferase-labeled dUTP were added, and the sections were incubated at 37°C for 1 h. Horseradish peroxidase-labeled specific antibody was added, and the sections were incubated again at 37°C for 1 h. After the nuclei were stained with 4',6'-diamidino-2-phenylindole(DAPI), images were collected using a fluorescence microscope. We chose and analyzed five fields in each section.

2. Supplementary Tables

Table S1. Composition and nutrient levels of the basal diet (air-dry basis).

ingredient	Level (%)
Alfalfa meal	62.80
Corn	15.99
Soybean meal	6.51
Wheat bran	6.11
Greaves	4.08
Premix	4.00
$NaHCO_3$	0.09
Allicin	0.10
NaCl	0.32
**nutrients**	**concentration**
DE/ (MJ/kg)	10.35
CP（%）	17.52
CF（%）	15.56
Ca（%）	0.83
P（%）	0.41

DE: digestible energy; CP: crude protein; CF: crude fiber; Ca: calcium; P: phosphorus.

Premix provided the following per kg of the diet: vitamin A – 8,000IU; vitamin B1 - 1.8mg; vitamin B2 - 6mg; vitamin B6 - 0.3mg; vitamin D - 800IU; vitamin E 50 mg; Cu 50mg; Fe 100mg; Zn 50mg; Mn 30mg; Mg 150mg; Se 0.1mg.

Table S2. The sequence of the primers used to analyze gene expression in Rex rabbits.

Gene	Primer sequence (5′-3′)	Product size/bp
FOSL1	F:AGCATCAACGCTGTGAGTGG<br>R:CTTCCTCTGGGCTGATCTGTT	196
IL1A	F:GGAAATGCCTGAGACACCCA<br>R:GCCATGTGCACCAGATGTTC	150
AXIN2	F:ATGCCTGTGTCGTCCAACG<br>R:AGGGATTCCATCTACGCTGC	713
HMGCS2	F:TAGTGCAACGGCTGATGGAG<br>R:GCTGGCTGACAGGAAGTCAT	647
JUN	F:AAACAGAGCATGACCCTGAACC<br>R:GGTTCCTCATGCGCTTCCTCT	703
FZD2	F:AGACCATCATGCCCAACCTG<br>R:ACCAGGTGAGGATCCAGAGG	594
RSPO1	F:AAAGACCGTCACTGGGTTGG<br>R:CTTGCATTCTGCGGAGAGGA	631
FGF18	F:GGGGACAAGTATGCCCAGCTC<br>R:CCACGTACCAGCCGGAGTA	217
PDGFRB	F:GATCAACAGGGAGGAGACGG<br>R:GAAGCTCATGGTGGGATCTGG	739
DUSP1	F:CCATCAGAAATGCCGGAGGA<br>R:CCAGCACCTGCGATTCAAAC	204
DUSP5	F:GCAAGGTCCTGGTTCACTGT<br>R:CGAAGGCAGGATCTCGGATT	191
FOS	F:CTACTACCACTCCCCAGCCG<br>R: TTCTAGCTGATCTGTCTCCGCCT	436
NGF	F:CCACATGGAGAGTTCTCGG<br>R:TCGGCACTTGGTCTCGAAAA	542
KRT23	F:GAAATCACCACCTACCGCCA<br>R:CACCCGTTAGTTTCTGGGCA	243
MSX2	F:ATGGCTTCTCCGTCCAAAGG<br>R: GGACTCATGTGTCTGGGTGG	395
IL1R2	F:ATGGAGGATGCGGGCTACTA<br>R:AAGCTGATATGGTCTGGCGG	151
THBS2	F:TTACGTCAACACTGACCGGG<br>R:GAGTTCACCACCTTGAGCGA	168
BMP3	F:GCCAGACACTCCAGTTCGAT<br>R:ACCCCCACAGCTCTCACTAT	247
SFRP1	F:GTCTCACGTCAGCCAGTTCA<br>R:AGCGAGCTGTAACAACCTCC	167
FGF12	F:CGAAGGACGAAAACAGCGAC<br>R:GCTGACGGTACAGTGTGGAA	213
TGFβ1	F:CCCTACATCTGGAGCCTGGAC<br>R:CTTTCACCTTTAATAGCCCGCA	339
ACTB	F:CACCCTCTCTCGACGAAACC<br>R:CAATCAAAGTCCTCGGCCAC	217

Table S3. Differential metabolites of rabbits in the MT and CR groups.

Metabolite name	variable importance in projection (VIP)	Log2FC	P-value	Ion mode	Expression Pattern
(R)-lipoic acid	1.588101	-1.26657	0.000709	pos	down
3-(3,4-Dimethoxyphenyl)-2-propenoic acid	1.63607	3.411386	1.23E-05	pos	up
1-(4-Methoxyphenyl)-2-propanone	1.57078	3.158866	0.000215	neg	up
15(S)-HPETE	1.390679	-1.8607	0.006814	neg	down
1-Methylhistidine	1.426713	-1.51264	0.016406	neg	down
1-Naphthol	1.634897	3.11412	0.000478	pos	up
LysoPC(18:1(9Z))	1.455517	-2.65966	0.010944	pos	down
2,4-Dihydroxybenzoic acid	1.451059	-1.34716	0.006268	neg	down
Aminoadipic acid	1.254917	-2.6717	0.03968	pos	down
2-Hydroxyadenine	1.469399	1.325499	0.008275	pos	up
3-(3,4,5-Trimethoxyphenyl)propanoic acid	1.618663	3.863977	0.001401	pos	up
4-Ethylbenzaldehyde	1.646359	4.276476	1.76E-05	pos	up
Isocaproic acid	1.345803	-1.18961	0.000491	neg	down
4-Oxoproline	1.514682	1.909113	0.00274	neg	up
4-Pyridoxic acid	1.321925	-1.38198	0.009069	neg	down
5,15-DiHETE	1.530203	-2.46424	0.018678	neg	down
Scopoletin	1.495983	3.91674	0.037114	pos	up
Nutriacholic acid	1.557935	-1.99211	0.003348	pos	down
7-Methylguanine	1.607024	1.745159	0.000673	pos	up
8,15-DiHETE	1.240706	1.545857	0.040738	pos	up
Alternariol	1.554025	2.58033	0.000527	pos	up
Atropine	1.538157	2.119329	0.000795	pos	up
NADP	1.360769	2.495646	0.013421	pos	up
Biotin	1.507623	1.805725	0.002748	pos	up
Cholesterol sulfate	1.474928	-2.05827	0.005724	neg	down
Cholic acid	1.353507	-3.97715	0.001617	neg	down
trans-Dec-2-enoic acid	1.001753	1.715737	0.009867	neg	up
cis-Aconitic acid	1.468076	-1.37951	0.011502	neg	down
Corchorifatty acid F	1.390885	-1.32053	0.007029	neg	down
Cytidine monophosphate	1.37062	-1.36136	0.014578	neg	down
Cytosine	1.521893	1.566141	0.004522	pos	up
D-Fructose	1.372514	-1.29915	0.037292	neg	down
D-Maltose	1.38198	-2.05634	0.016757	pos	down
D-Alanyl-D-alanine	1.261019	-2.51421	0.043772	pos	down
dCDP	1.427082	-1.9253	0.029938	neg	down
Capric acid	1.176106	1.92445	0.004006	neg	up
Deoxycholic acid	1.520887	-3.54449	0.000644	neg	down
Deoxycorticosterone	1.574298	1.908675	0.000254	pos	up
Glucosamine 6-phosphate	1.468762	-2.22343	0.009444	neg	down
Marindinin	1.402575	-1.11372	0.007427	pos	down

Malic acid	1.268005	-1.42144	0.038441	neg	down
D-Serine	1.436262	1.175352	0.001251	pos	up
Docosahexaenoyl Ethanolamide	1.564699	1.892186	0.001623	pos	up
Docosapentaenoic acid	1.301699	1.71751	0.031415	pos	up
Dodecanedioic acid	1.357672	2.364474	0.01137	neg	up
D-Proline	1.351324	-1.01139	0.013133	neg	down
Galactitol	1.451718	-1.6145	0.006549	neg	down
Equol	1.585773	-2.22181	0.001683	neg	down
Ethyl tetradecanoate	1.472418	1.349783	0.013309	neg	up
gamma-Glutamylleucine	1.60223	1.609899	0.000376	pos	up
Glycocholic acid	1.438365	-1.67385	0.009265	pos	down
Deoxycholic acid glycine conjugate	1.576902	-2.2358	0.000728	neg	down
Glycoursodeoxycholic acid	1.512806	-2.65263	0.011741	neg	down
Guanosine	1.388391	1.751659	0.016638	neg	up
Guggulsterone	1.242766	1.85985	0.033347	pos	up
Hexanoylcarnitine	1.088901	1.952426	0.033767	pos	up
Isoniazid	1.603799	3.602522	0.0061	pos	up
13-cis-Retinoic acid	1.397985	-1.18284	0.040754	pos	down
Kanosamine	1.602791	1.557157	0.000127	pos	up
L-Arabinose	1.292537	-3.42449	0.023183	neg	down
Argininosuccinic acid	1.269972	-1.31486	0.024409	neg	down
Dodecanoic acid	1.505486	1.021539	0.001786	neg	up
Ethyl dodecanoate	1.373146	1.472081	0.016017	neg	up
L-Histidine	1.34356	1.13705	0.014264	pos	up
Lithocholic acid	1.599449	-3.72899	0.000102	neg	down
L-Lysine	1.584897	-1.59244	0.005205	neg	down
L-Palmitoylcarnitine	1.48928	1.426481	0.001394	pos	up
L-Pipecolic acid	1.589163	-2.72341	0.005103	neg	down
Pyroglutamic acid	1.580106	2.464776	0.00041	pos	up
Saccharopine	1.393892	-1.55442	0.012696	neg	down
L-Serine	1.443885	1.396907	0.041131	neg	up
Maltotriose	1.447938	-2.44177	0.018751	neg	down
Melanin	1.349913	-1.07893	0.028492	pos	down
N2,N2-Dimethylguanosine	1.609608	1.668363	0.000592	pos	up
N4-Acetylcytidine	1.631288	2.283225	4.84E-06	pos	up
N-Acetyl-L-phenylalanine	1.56946	-1.92548	0.000832	neg	down
N-Acetylornithine	1.626627	-1.37204	1.64E-05	neg	down
Orlistat	1.557634	2.188835	0.000443	pos	up
4-Hydroxyproline	1.177036	-1.31129	0.049965	pos	down
Palmitoylcarnitine	1.547922	2.2006	0.002426	pos	up
Phenylacetylglycine	1.286577	1.50278	0.043465	pos	up
Pipecolic acid	1.609845	-3.15067	0.000131	neg	down
Porphobilinogen	1.461314	1.12378	0.026766	pos	up
Pregnenolone	1.449813	-1.41732	0.01018	pos	down
Propionylcarnitine	1.391574	-1.11572	0.029059	pos	down
Prostaglandin B2	1.385951	1.973941	0.023968	pos	up
Neuroprotectin D1	1.391378	2.666325	0.039316	neg	up

Pyridostigmine	1.628879	3.126775	3.32E-05	pos	up
NADH	1.287298	-1.15126	0.037035	neg	down
Salicylic acid	1.427663	-1.4724	0.00967	neg	down
Spermidine	1.349068	1.070198	0.030951	pos	up
Spermine	1.36946	3.160095	0.021836	pos	up
L-Urobilin	1.359927	-1.67532	0.027603	pos	down
Taurochenodesoxycholic acid	1.513313	-1.5895	0.024399	neg	down
Taurocholic acid	1.354755	-1.58046	0.031472	neg	down
Taurodeoxycholic acid	1.438651	-1.76758	0.023098	pos	down
Dronabinol	1.622713	3.951691	0.00028	pos	up
Traumatic acid	1.612564	-1.56075	0.000845	neg	down
Ursodeoxycholic acid	1.632244	-2.65609	0.000291	neg	down
17a-Estradiol	1.582633	-2.49888	0.000478	pos	down
16-Hydroxy hexadecanoic acid	1.183181	0.669055	0.038618	neg	up
1-Methylguanine	1.040559	0.475284	0.029837	pos	up
Xanthine	1.259001	0.72322	0.029484	pos	up
2-Furoic acid	1.393873	-0.84178	0.013215	neg	down
2-Hydroxymyristic acid	1.416386	-0.8562	0.011127	neg	down
3-Furoic acid	1.501516	-0.89174	0.00294	neg	down
3-Hydroxyisovaleric acid	1.517406	-0.66702	0.001039	pos	down
3-Methylhistidine	1.26326	-0.85209	0.033484	pos	down
5'-Methylthioadenosine	1.089336	-0.40164	0.034748	pos	down
5-Hydroxyindoleacetic acid	1.432172	0.802069	0.03527	pos	up
5-Hydroxylysine	1.169538	-0.8013	0.042731	pos	down
5-Hydroxy-L-tryptophan	1.441862	-0.88463	0.013551	neg	down
Acetoacetic acid	1.338058	-0.79323	0.009991	neg	down
L-Acetylcarnitine	1.218583	-0.67319	0.015827	pos	down
Oxoglutaric acid	1.468785	-0.82548	0.004543	neg	down
Azelaic acid	1.353547	-0.85145	0.016311	neg	down
Citrulline	1.38225	0.775784	0.017088	neg	up
Citicoline	1.30485	0.890295	0.023188	pos	up
dCMP	1.280818	-0.64158	0.026621	neg	down
D-Erythrose 4-phosphate	1.139287	-0.52789	0.020287	neg	down
Elaidic acid	1.353769	0.751449	0.019155	neg	up
Epicatechin	1.308402	-0.6438	0.02228	pos	down
Ergothioneine	1.191468	0.894442	0.038892	pos	up
Glycerol 3-phosphate	1.160314	-0.7945	0.042134	pos	down
Heptadecanoic acid	1.296073	0.939617	0.006721	pos	up
Hydrocinnamic acid	1.179423	-8.26287	0.024988	neg	down
Kojic acid	1.30986	-0.69905	0.0198	neg	down
Alanyl-Lysine	1.196672	-0.66845	0.021052	neg	down
L-Aspartic acid	1.319504	-0.8109	0.027266	neg	down
L-Threonine	1.371552	0.678216	0.015715	pos	up
Nicotinic acid	1.404862	-0.93251	0.007731	neg	down
Norepinephrine	1.506755	-0.68061	0.001091	pos	down
Phenylacetaldehyde	1.211993	-0.68022	0.040348	neg	down
Phenylpyruvic acid	1.443273	-0.87073	0.004403	neg	down

Phytosphingosine	1.36308	0.576426	0.013094	pos	up
Prostaglandin G2	1.280682	1.02208	0.038429	pos	up
Pyridoxamine	1.575719	-0.96969	0.000617	pos	down
Stearic acid	1.376294	0.600812	0.021669	neg	up
Thioguanine	1.088609	-0.69595	0.045487	neg	down
Thymidine	1.301254	-0.75475	0.023832	neg	down
Tridecanoic acid	1.281648	0.544703	0.006037	neg	up
Tyramine	1.015226	-0.42216	0.01204	pos	down
Uridine 5'-diphosphate	1.610251	0.950371	8.19E-05	pos	up
D-Xylitol	1.491309	-0.91025	0.003726	neg	down

3. Supplementary Figures

CR group

MT group

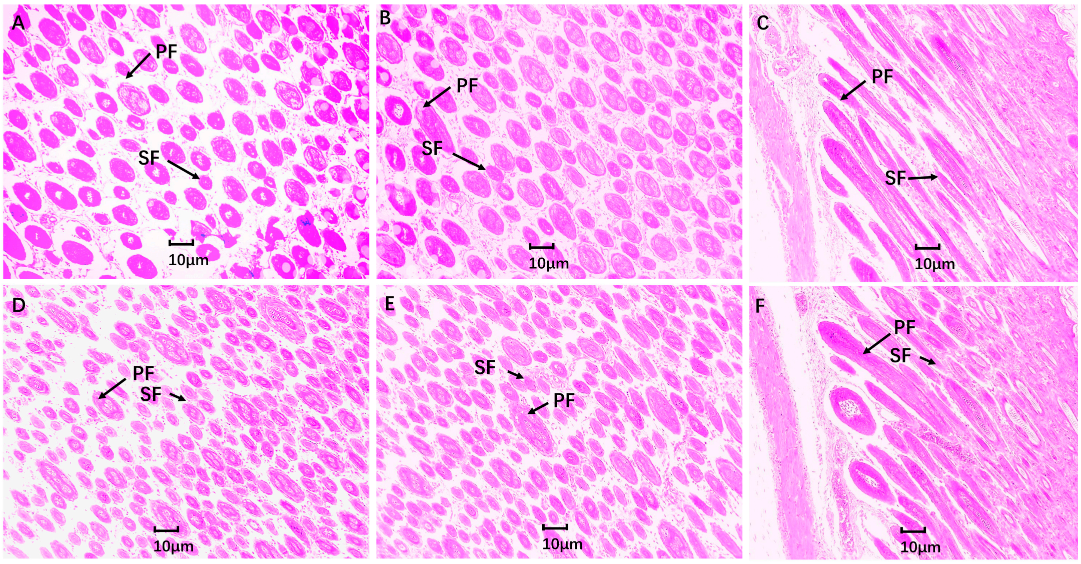

Figure S1. Effects of maternal melatonin supplementation on the morphology of hair follicle in 1-day-old Rex rabbit offspring. (A, B, D, E) representative hair follicle groups at horizontal sections; (C, F) representative hair follicles at longitudinal sections. (A, B, C) are the skin of kids in control group at the age of 1-day-old; (D, E, F) are the MT group [25].

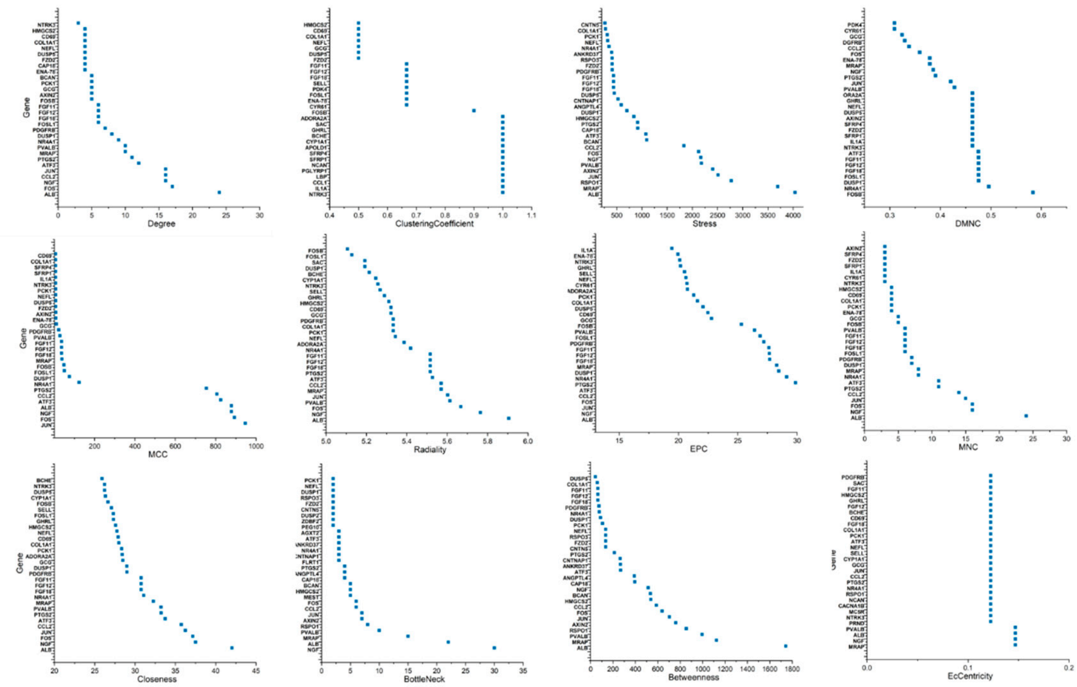

Figure S2. The top 30 genes and scores with different algorithms rank.

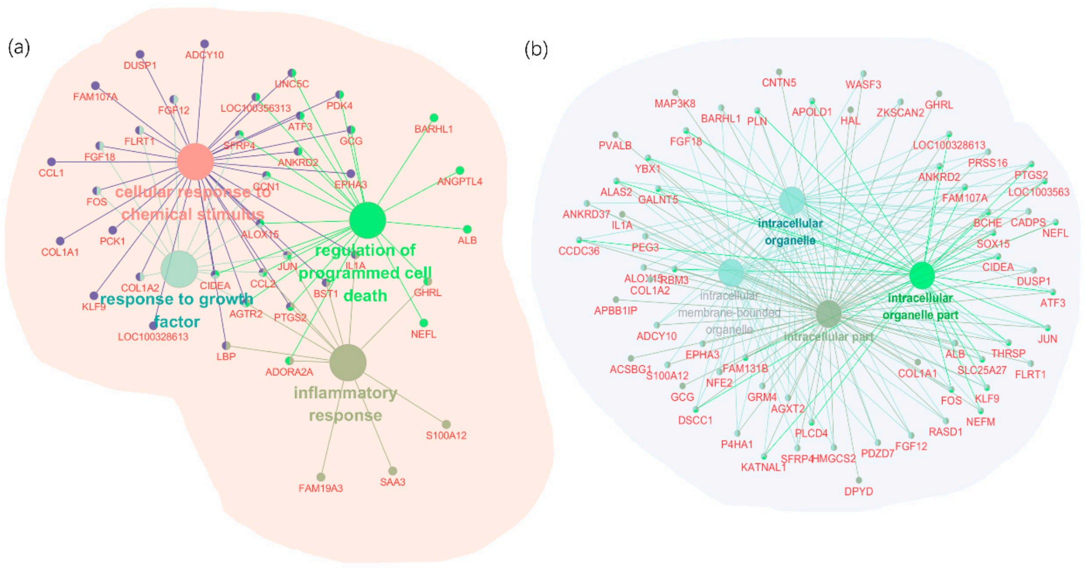

Figure S3. Enriched GO terms of DEGs at Class-up profile. (a) Biological Processes (BP); (b) Cellular Component (CC)

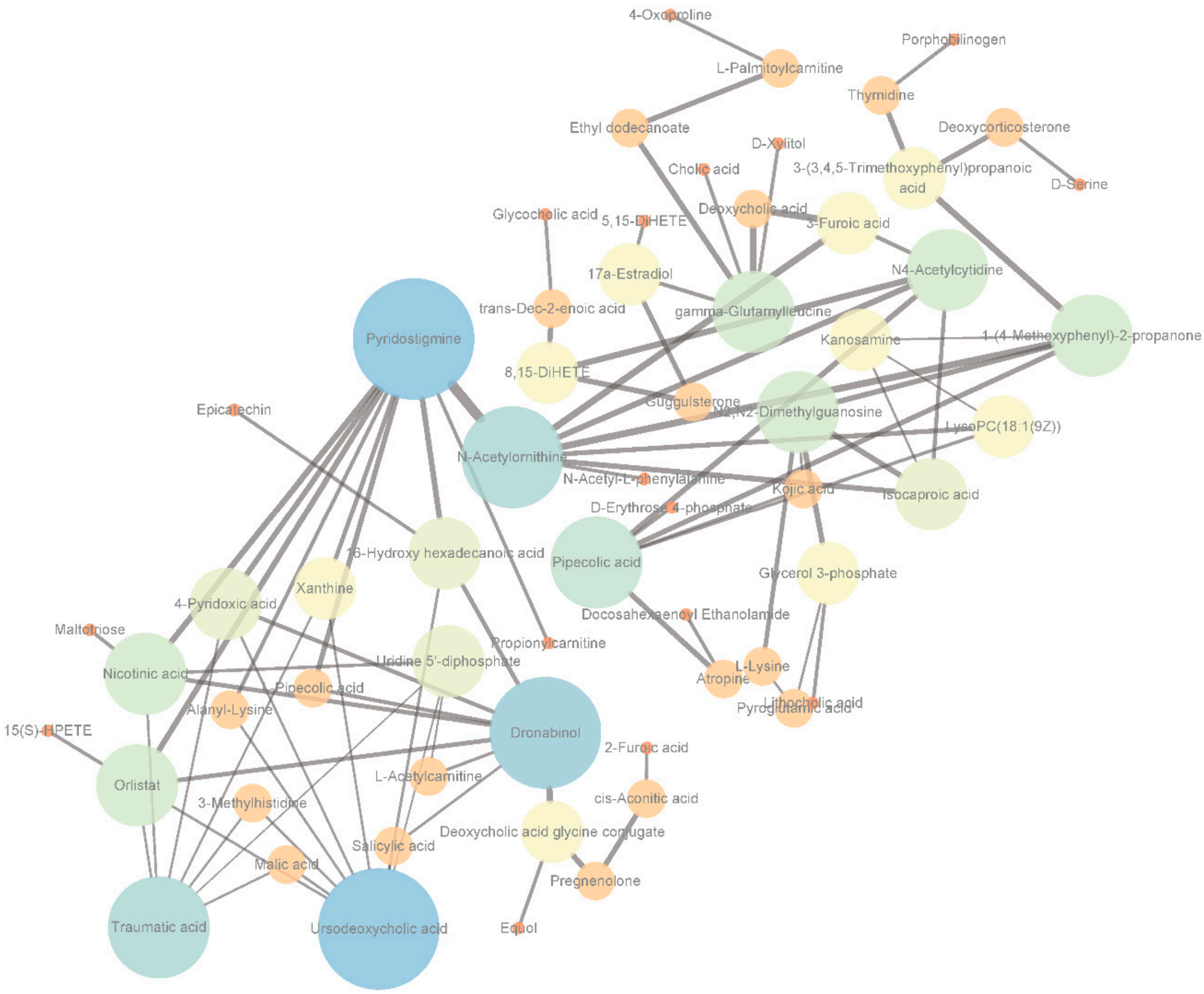

Figure S4. The DSPC network of the skin different metabolites for group MT vs. CR. The size of the circular node represents the importance of the current metabolite in the reciprocal network, and the thickness of the connecting line indicates the degree of correlation between the two metabolites. As the DSPC network shows, Pyridoxamine, N-Acetylornithine, Ursodeoxycholic acid, Traumatic acid, Dronabinol, Pipecolic acid, Nicotinic acid, 4-Pyridoxic acid, N4-Acetylcytidine,1-(4-Methoxyphenyl)-2-propanone, gamma- Glutamylleucine at key nodal positions, interacting effects with multiple metabolites.

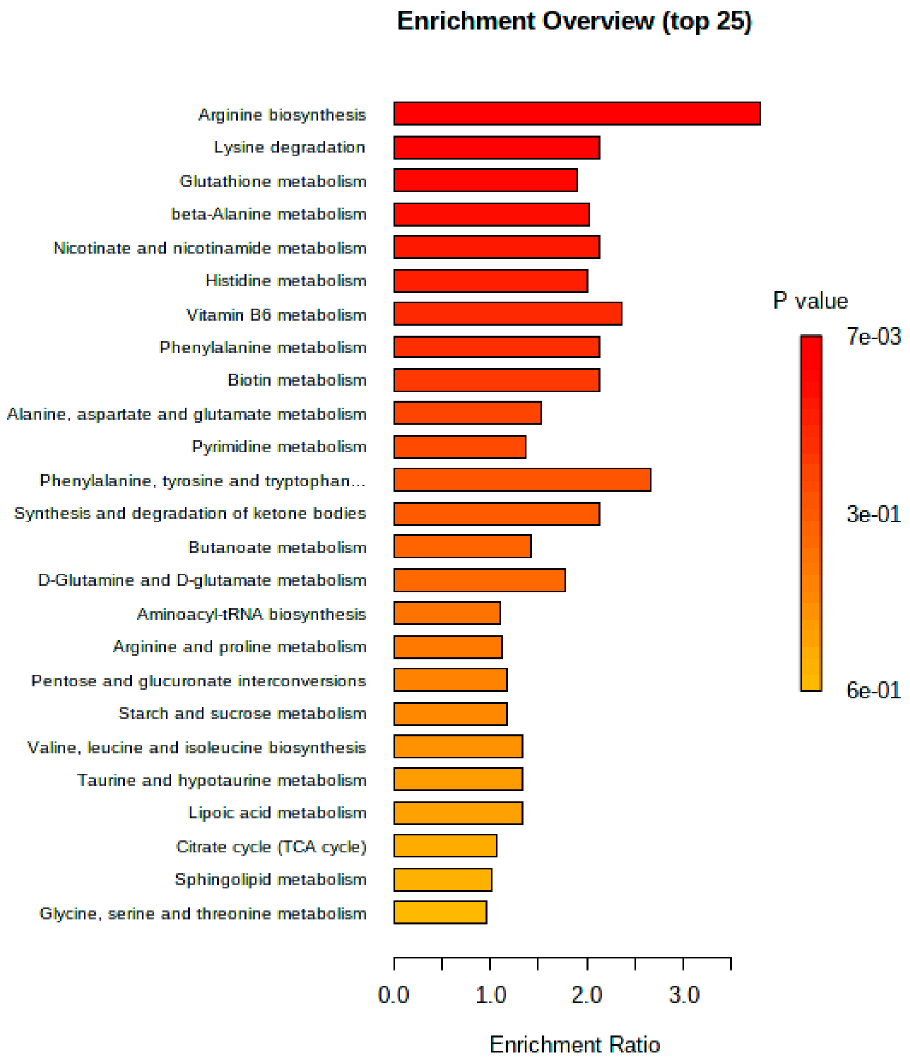

Figure S5. The top 25 significantly enriched KEGG pathways of the metabolites in MT and CR group. The DEMs were mapped followed the KEGG database and a total of 46 metabolic pathways were mapped, with the top 25 significant pathways plotted in the figure. The DEMs were most enriched in amino acid-related metabolic pathways and B-vitamin-related metabolic pathways.

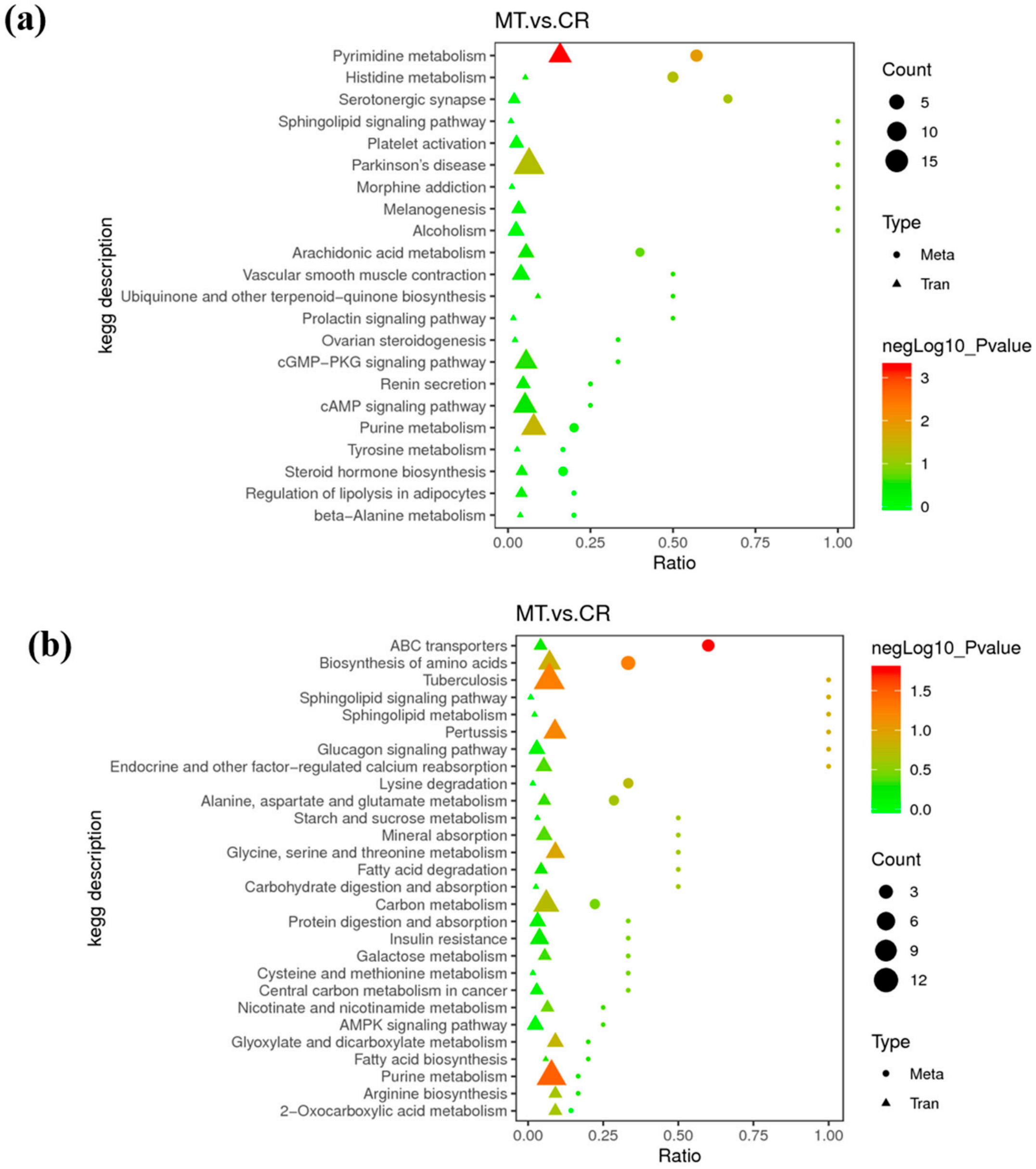

Figure S6. (a) Correlation analysis of KEGG pathway between differential genes and metabolites in positive ion modes;(b) Correlation analysis of KEGG pathway between differential genes and metabolites in negative ion modes. The horizontal coordinate is the ratio of differential metabolites or differential genes enriched in the pathway to the number of metabolites or genes annotated in the pathway (Ratio), and the vertical coordinate is the KEGG pathway that the metabolome and transcriptome are jointly enriched to. Count: the number of metabolites or genes enriched in the pathway. Analysis of the common pathways enriched to DEGs and DEMs in amino acid-related pathways, endocrine hormone-related pathways, and nutrient-related pathways in the positive ion mode.

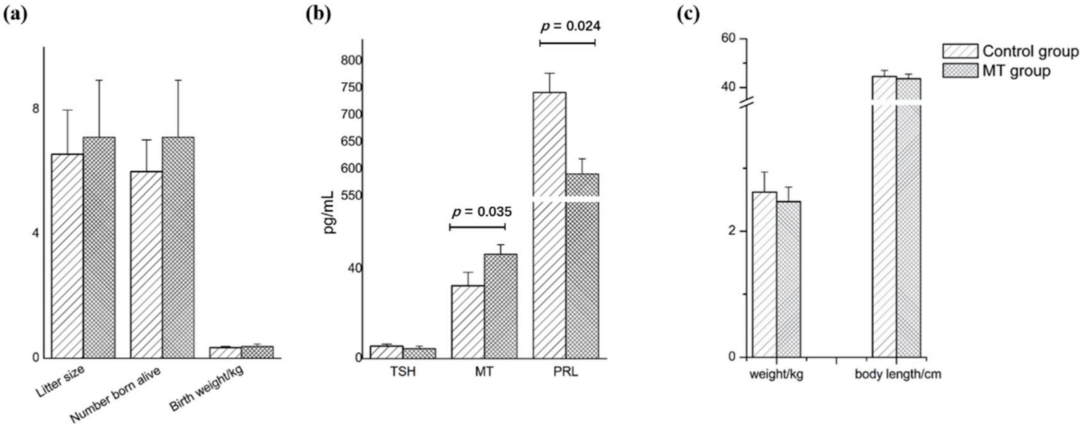

Figure S7. a): Reproductive performance of female rabbits with and without MT; (b):The concentrations of maternal serum biochemical parameters in rabbits with and without MT implantation; (c): Body weight and body length of 5-months-old rabbits. [25].

No p values label means no significance ($p > 0.05$).

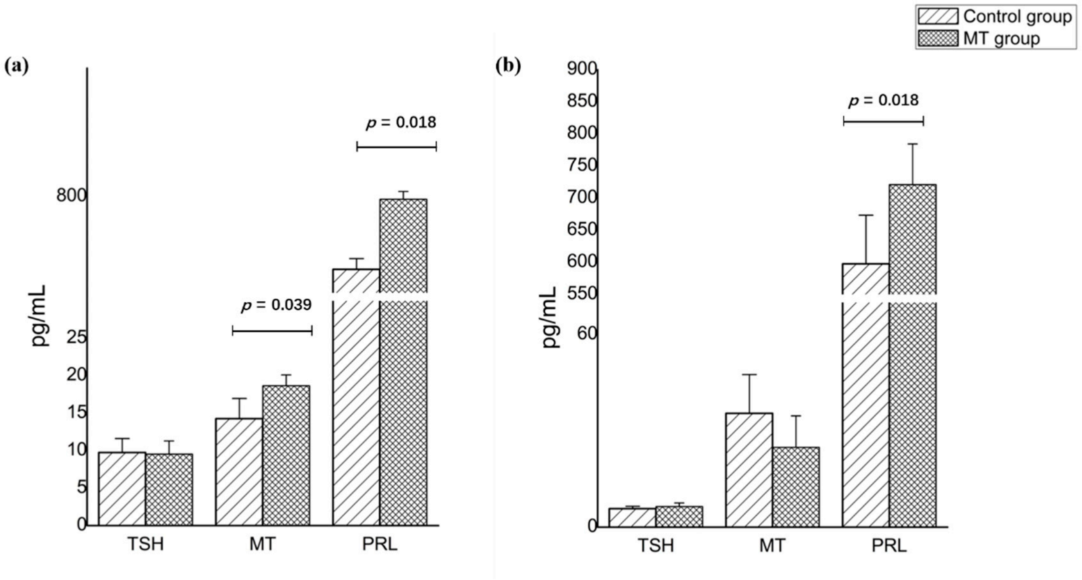

Figure S8. The concentrations of TSH, PRL, and MT in the serum of first-generation rabbits. (a): 1-day old; (b): 5 months old. [25]

References

25. Feng, Y.; Gun, S. Melatonin supplement induced the hair follicle development in offspring rex rabbits. *J. Anim. Physiol. Anim. Nutr.* **2020**, *105*, 167–174, https://doi.org/10.1111/jpn.13417.
58. Chin, C.-H.; Chen, S.-H.; Wu, H.-H.; Ho, C.-W.; Ko, M.-T.; Lin, C.-Y. cytoHubba: identifying hub objects and sub-networks from complex interactome. *BMC Syst. Biol.* **2014**, *8* (Suppl. 4), S11, doi:10.1186/1752-0509-8-s4-s11.
59. Kleinpenning, F.; Steigenberger, B.; Wu, W.; Heck, A.J.R. Fishing for newly synthesized proteins with phosphonate-handles. *Nat. Commun.* **2020**, *11*, 1–10, https://doi.org/10.1038/s41467-020-17010-0.
60. Saccenti, E.; Hoefsloot, H.C.J.; Smilde, A.K.; Westerhuis, J.A.; Hendriks, M.M.W.B. Reflections on univariate and multivariate analysis of metabolomics data. *Metabolomics* **2013**, *10*, 361–374, https://doi.org/10.1007/s11306-013-0598-6. .
61. Minoru K, Yoko S, Masayuki K, Miho F, Mao T. KEGG as a reference resource for gene and protein annotation. Nucleic Acids Research. 2016(D1):D457-D62.
